# The Frailty Trajectory’s Additional Edge Over the Frailty Index: Retrospective Cohort Study of Veterans With Heart Failure

**DOI:** 10.2196/56345

**Published:** 2024-06-27

**Authors:** Javad Razjouyan, Ariela R Orkaby, Molly J Horstman, Parag Goyal, Orna Intrator, Aanand D Naik

**Affiliations:** 1 Baylor College of Medicine, Houston, TX, United States; 2 VA Health Services Research & Development, Center for Innovations in Quality, Effectiveness and Safety, Michael E. DeBakey VA Medical Center, Houston, TX, United States; 3 Big Data Scientist Training Enhancement Program, VA Office of Research and Development, Washington, DC, United States; 4 New England Geriatrics Research, Education, and Clinical Center, VA Boston Health Care System, Boston, MA, United States; 5 Brigham & Women's Hospital, Harvard Medical School, Boston, MA, United States; 6 Division of General Internal Medicine, Department of Medicine, Weill Medical College of Cornell University, New York, NY, United States; 7 Geriatrics and Extended Care Data Analysis Center, Canandaigua VA Medical Center, Canandaigua, NY, United States; 8 Public Health Sciences, University of Rochester School of Medicine and Dentistry, Rochester, NY, United States; 9 Department of Management, Policy, and Community Health, School of Public Health, University of Texas Health Science Center, Houston, TX, United States; 10 Institute on Aging, University of Texas Health Science Center, Houston, TX, United States

**Keywords:** gerontology, geriatric, geriatrics, older adult, older adults, elder, elderly, older person, older people, ageing, aging, frailty, frailty index, frailty trajectory, frail, weak, weakness, heart failure, HF, cardiovascular disease, CVD, congestive heart failure, CHF, myocardial infarction, MI, unstable angina, angina, cardiac arrest, atherosclerosis, cardiology, cardiac, cardiologist, cardiologists

## Introduction

Individuals with heart failure (HF) have a high burden of health care utilization, costs, and morbidity in the year following hospitalization for an acute HF exacerbation. Frailty, which has been described as increased vulnerability to adverse events, is common among those with HF and increases with age [1]. Health systems worldwide are integrating automated tools within electronic health records to measure frailty. However, using longitudinal data to measure frailty and better predict outcomes among those with HF has rarely been considered [2-5]. We sought to evaluate the predictive value of adding longitudinal data to a standard frailty index (FI) and evaluate predictions of 1-year outcomes in patients with HF.

## Methods

### Study Design

This was a retrospective cohort study that used national Veterans Health Administration (VA) data. Veterans aged ≥50 years with an index hospital admission for HF from 2016 to 2019 were included. We excluded veterans with <2 primary care visits in the 3 years before their date of admission to indicate regular use of VA care. We included those with documentation of ejection fraction. We used the validated VA FI, which captures 31 deficits in health based on *International Classification of Diseases, Tenth Revision*, and Current Procedural Terminology codes [6]. We estimated the FI for each preceding year, without overlap. We fit a linear line to 3 calculated FIs for each year prior to the index date of admission and reported the slope and intercept individually. This method provided a 3-year longitudinal estimate of frailty at admission. We used 1-year all-cause mortality following the index date of admission as the primary outcome. We reported the area under the curve (AUC) for predicting outcomes, using logistic regression. We estimated two AUCs: (1) FI at the time of admission (AUC_FI_) and (2) FI at time of admission plus slope and intercept (AUC_frailty trajectory (FT)+FI_). Changes in the AUCs were reported as the percentage of improvement (Δ_AUC_ = 100% × [AUC_FT+FI_ – AUC_FI_]/AUC_FI_). We recursively calculated the AUCs and Δ_AUC_ by including patients whose FIs at admission were <0.1 and, at each step, increased the FI level by 0.01 to 0.4.

### Ethical Considerations

The study protocol was approved by the Research & Development Committee of the Michael E. DeBakey VA Medical Center and Baylor College of Medicine Institutional Review Board (institutional review board number: H-464220).

## Results

**Figure 1.**
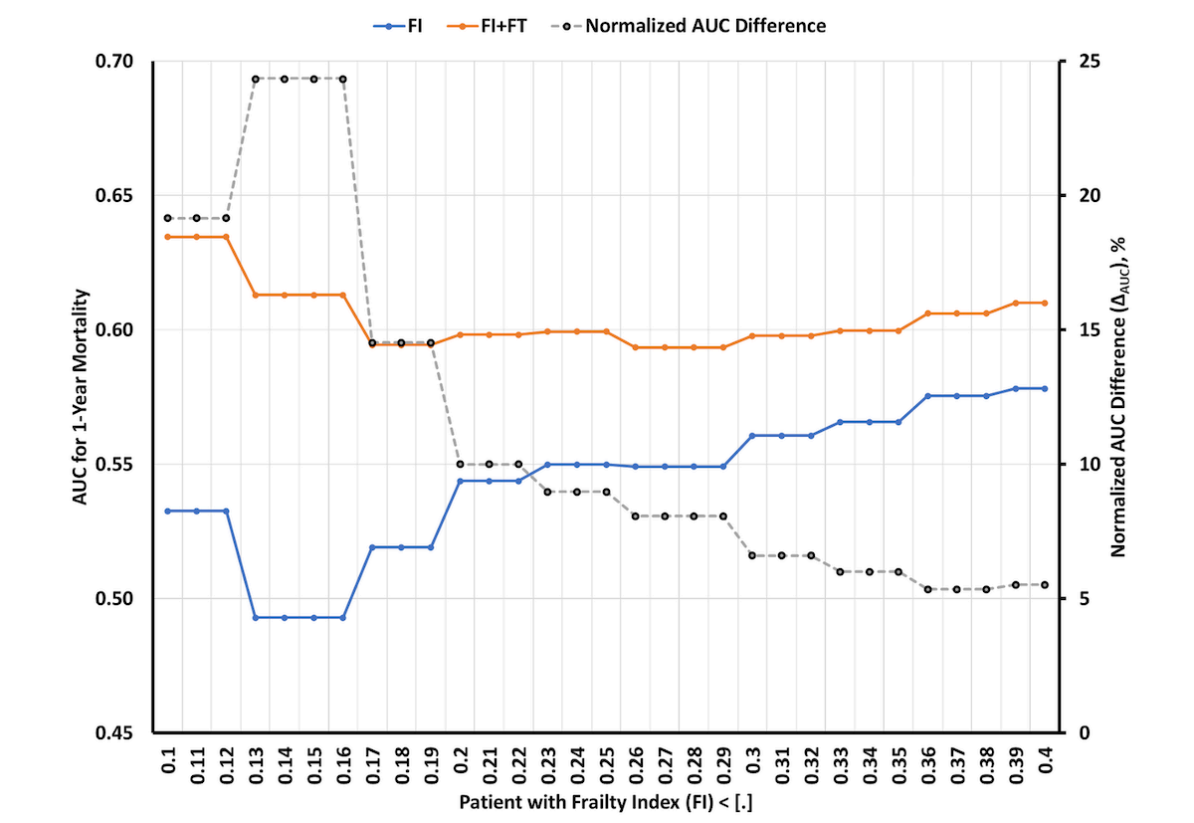
AUCs for patients who were admitted, for the first time, to the Veterans Health Administration for heart failure from January 1, 2016, to January 1, 2020, and had an FI of 0.1-0.4 (as shown on the x-axis in increments of 0.01). We compared the AUCs of FIs (in blue; AUC_FI_) versus the AUCs of FIs and FTs combined (in orange; AUC_FI+FT_). The percentage of improvement in AUCs resulting from the addition of the FT to the FI was reported in black (Δ_AUC_) and calculated by using the following formula: $\Delta_{AUC}=\;\frac{\left(AUC_{FI+FT}-AUC_{FI}\right)}{AUC_{FI}}\;\times 100$. AUC: area under the curve; FI: frailty index; FT: frailty trajectory.

In total, 54,774 veterans were included (age: mean 73.3, SD 10.1 y; BMI: mean 30.1, SD 7.5 kg/m^2^; male: n=53,899, 98.4%; White: n=30,406, 55.5%; Table 1). Figure 1 shows the AUC_FI_ and AUC_FT+FI_ across the distribution of frailty ranges, from prefrail (FI: 0.1-0.2) to frail; an FI of 0.2 is equivalent to an accumulation of 7 deficits among 31 variables, and the Δ_AUC_ is also displayed. For all veterans across all FI thresholds, the AUC improved by at least 4.1% when adding the FT to the FI. The highest Δ_AUC_ (24%) was observed for FIs of 0.13 to 0.16, and it decreased to ≤10% for FIs of ≥0.2.

**Table 1.** Characteristics of patients (N=54,774) with an index admission to the Veterans Health Administration for heart failure from January 1, 2016, to January 1, 2020.

Characteristics		Patients
Admit year 2016, n (%)		12,875 (23.5)
Admit year 2017, n (%)		13,585 (24.8)
Admit year 2018, n (%)		14,082 (25.7)
Admit year 2019, n (%)		14,232 (26)
**Age (y), mean (SD)**		73.3 (10.1)
	<65, n (%)	9776 (17.8)
	65‐75, n (%)	22,772 (41.6)
	≥85, n (%)	22,226 (40.6)
**Sex, n (%)**		
	Male	53,899 (98.4)
	Female	875 (1.6)
**Race, n (%)**		
	White	30,406 (55.5)
	Black	9340 (17.1)
	Other^a^	15,028 (27.4)
Hispanic ethnicity, n (%)		2093 (3.8)
**BMI (kg/m** ^**2**^ **), mean (SD)**		30.1 (7.5)
	≥30, n (%)	24,352 (44.5)
**Frailty status (frailty index), mean (SD)**		0.35 (0.11)
	Robust (<0.1), n (%)^b^	297 (0.5)
	Prefrail (0.1‐0.2), n (%)^b^	5715 (10.5)
	Frail (>0.2), n (%)^b^	48,762 (89)
**All-cause mortality, n (%)**		
	30-day mortality	2848 (5.2)
	1-year mortality	14,460 (26.4)
	All-time mortality	37,027 (67.6)
Time to death (mo), median (IQR)		18.2 (5.6-36.4)
HFrEF^c^, n (%)		27,223 (49.7)
HFmEF^d^, n (%)		4546 (8.3)
HFpEF^e^, n (%)		23,005 (42.0)
Living in a CLC^f^, n (%)		1808 (3.3)

a “Other” includes Asian, American Indian or Alaska Native, Native Hawaiian or other Pacific Islander, and unknown.

b Standardized frailty status cut points drawn from validated studies [6].

c HFrEF: heart failure with reduced ejection fraction of <40%.

d HFmEF: heart failure with modified reduced ejection fraction of 40%-50%.

e HFpEF: heart failure with preserved ejection fraction of >50%.

f CLC: community living center.

## Discussion

In a national cohort of veterans who were admitted to the VA for HF, the addition of longitudinal FT data resulted in a clinically significant (up to 24%) improvement in 1-year mortality prediction when compared to a standard FI alone among patients in the prefrail range. In contrast, we observed a modest (at least 4.1%) improvement in 1-year mortality prediction in the overall population. Enhancing AUC prediction for patients in the prefrail range is clinically important, as interventions that mitigate frailty may be most impactful in this population [7]. Patients with prefrailty may benefit from interventions (eg, cardiac rehabilitation) that improve frailty status and cardiovascular outcomes [1]. These findings enrich our understanding of the importance of FT in patients at lower FI levels, and a previous study compared the importance of FIs to that of FTs alone [5]. These results may not generalize to nonveteran populations. The sample was predominately male but did include a diverse population in terms of race, ethnicity, and geographic distribution. In summary, methods for calculating frailty provide useful predictions of adverse outcomes among adults with HF. The addition of longitudinal frailty data improves predictions for patients with HF and prefrailty. These findings aid clinician and health system decision-making, as this population benefits most from interventions that slow or prevent frailty progression, and suggest that longitudinal data for modeling FT provide additional evidence for tailoring interventions to patients with HF who may benefit most from tailored interventions.

## Abbreviations

AUC: area under the curve
FI: frailty index
FT: frailty trajectory
HF: heart failure
VA: Veterans Health Administration
